# Impact of organizational climate on organizational commitment and perceived organizational performance: empirical evidence from public hospitals

**DOI:** 10.1186/s12913-018-3149-z

**Published:** 2018-06-01

**Authors:** Aysen Berberoglu

**Affiliations:** Faculty of Economics and Administrative Sciences, Cyprus International University, Haspolat, Nicosia, Cyprus

**Keywords:** Public hospitals, Organizational performance, Climate, Commitment, Management, North Cyprus

## Abstract

**Background:**

Extant literature suggested that positive organizational climate leads to higher levels of organizational commitment, which is an important concept in terms of employee attitudes, likewise, the concept of perceived organizational performance, which can be assumed as a mirror of the actual performance. For healthcare settings, these are important matters to consider due to the fact that the service is delivered thoroughly by healthcare workers to the patients. Therefore, attitudes and perceptions of the employees can influence how they deliver the service. The aim of this study was to evaluate healthcare employees’ perceptions of organizational climate and test the hypothesized impact of organizational climate on organizational commitment and perceived organizational performance.

**Methods:**

The study adopted a quantitative approach, by collecting data from the healthcare workers currently employed in public hospitals in North Cyprus, utilizing a self-administered questionnaire. Collected data was analyzed with the help of Statistical Package for Social Sciences, and ANOVA and Linear Regression analyses were used to test the hypothesis.

**Results:**

Results revealed that organizational climate is highly correlated with organizational commitment and perceived organizational performance. Simple linear regression outcomes indicated that organizational climate is significant in predicting organizational commitment and perceived organizational performance.

**Conclusions:**

There was a positive and linear relationship between organizational climate with organizational commitment and perceived organizational performance. Results from the regression analysis suggested that organizational climate has an impact on predicting organizational commitment and perceived organizational performance of the employees in public hospitals of North Cyprus. Organizational climate was found to be statistically significant in determining the organizational commitment of the employees. The results of the study provided some critical issues regarding the relationship of three concepts in the study. According to the findings, if the organizational climate scores of the employees are high, organizational commitment scores of the employees are high at the same time. In other words, if the employees in public hospitals of North Cyprus perceive the organizational climate in a positive way, they will have higher levels of organizational commitment. Findings suggested that organizational climate is an important factor in healthcare settings in terms of employee commitment and how employees perceive organizational performance, which would lead to significant results about the provision of service in healthcare organizations.

There has been increasing attention on the relationship between management and employees both in the business and in academic world since 1930s. Attitudes of the employees towards their organization, as a result of their work environment, are important issues in organizational behavior literature. Employee behavior in organizations is a result of their personal characteristics as well as the environment, which they work in. In this regard, organizational climate is an important aspect in order to understand employee’s work-related behavior and it has been discussed in organizational behavior literature since late 1960s.

Simply, organizational climate is the aggregate of psychological climates, which are the perceptions of individuals about their work environments [1]. However, defining the climate in an organization precisely is not easy because it is based on the perceptions of employees. Nevertheless, it is certain that the climate has a strong influence on employee attitudes regarding their sense of belonging, personal relationships and work performance [2]. Additionally, concepts such as job satisfaction, need for achievement, affiliation and power, overall organizational effectiveness and performance, and organizational commitment are found to be the consequences of perceived organizational climate [3]. Moreover, organizational commitment of employees, towards their organization, is found to have a significant relationship with and influence on the overall organizational performance. Lastly, individual employee performance is also found to be correlated with organizational commitment [4].

The aim of this study is to evaluate the relationship between organizational climate, organizational commitment and perceived organizational performance among health care professionals currently working in public hospitals of North Cyprus. The study aims to reveal the nature of relationship between the dependent and independent variables that are organizational climate (independent variable) on organizational commitment (dependent variable 1) and perceived organizational performance (dependent variable 2).

North Cyprus health system is comprised of both public and private institutions. State hospitals in North Cyprus are funded by the government. There are privately owned hospitals, one university hospital and privately owned clinics in the country. The largest public hospital is located in the capital, Nicosia, and the other public hospitals are located in three districts. In addition to these, there is a public hospital specialized on mental and neurological disorders in Nicosia. The health system in North Cyprus can be categorized as a blend of National Health Service System Model (NHS Model) and Social Insurance System, which were mentioned, by Kikuzawa, Olafsdottir, and Pescosolido [5]. In this respect, the government provides health care to the citizens through publicly owned hospitals and, residents have healthcare insurance when they are working or retire in Northern Cyprus to receive health care service free of charge. In the case of North Cyprus, it is important to evaluate and understand the behavioral motivations of the health care employees in public hospitals in order to realize how this motivation can have an impact on provision of healthcare services.

The present study is currently the one and only study aimed to address the relationship between organizational climate, organizational commitment and perceived organizational performance of health care workers in North Cyprus’ health care settings. There are previous studies conducted in public and private hospitals but they were not significantly able to generalize their findings in public health sector of North Cyprus. The study will contribute to the current literature by presenting empirical evidence about the influence of organizational climate on organizational commitment and perceived organizational performance with the use of perceptions of health care workers in public hospitals of North Cyprus. However, the previous literature involves comprehensive research regarding the relationship between these three concepts separately, and the current study contributes to the existing knowledge by analyzing the influence of organizational climate on organizational commitment and perceived organizational performance collectively, unlike the existing studies in the literature.

## Background

### Organizational climate

Employee behavior in organizations is a result of their personal characteristics as well as the environment in which they perform. Employees’ job attitudes are affected by a wide range of organizational characteristics and social relationships, which form the employees’ work environment [6]. When referring to employees’ perceptions of their working environments, it is possible to find a variety of terms and definitions such as organizational climate, psychological climate, collective climate, and organizational culture [7]. Organizational climate is one of the most important matters regarding organizational environment, which has a direct relationship with employee behavior. Since late 1960s, organizational climate has been a popular topic discussed in organizational behavior literature and is considered as a vital viewpoint in order to comprehend employee’s work-related attitudes and behaviors. Payne et al. [8] defined organizational climate as the way in which employees perceive their organization and its purposes. Churchill et al. conceptualized organizational climate as the aggregates of the social variables, which constitute a worker’s job environment [6]. According to Mullins [2], if organizational culture is defined simply as ‘how things are done around here’, then organizational climate can be defined as ‘how it feels to work around here’. Griffin and Moorhead explained organizational climate as individual perceptions; recurring patterns of behaviour, attitudes and feelings of employees [9].

Additionally, Robbins and Judge stated that organizational climate can be considered as an aspect of culture and defined as team spirit but at the organizational level [10], and according to Uhl-Bien et al. [11], one of the most important aspects in an organization to influence how people behave is organizational culture that can be defined as the shared beliefs and values within the organization [11]. In order to understand how an employee perceives organizational climate, it is necessary to consider the employee’s perceptions of the work situation (including the characteristics of the organization they work for) and the nature of his/her relationships with other people in the same environment [6]. Organizational climate has a significant impact on the well-being of employees that has a direct influence on quality and quantity of work done in the organization [2]. There are various studies regarding the relationship between organizational climate and its consequences. The concepts like job satisfaction, need for achievement, affiliation and power, overall organizational effectiveness and individual performance are found to be the dependent variables and consequences of organizational climate [3]. In addition, one of these consequences is organizational commitment, and a moderate level of attention is given in the literature to reveal this relationship. Consequently, the relationship between organizational climate and organizational performance is also a widely discussed topic, but perceived organizational performance is a relatively new concept and has not drawn the attention on itself yet. Permarupan et al. [12] suggested that organizational climate perceived by employees influences the motivation of employees and motivation will result in higher productivity so, a positive climate is said to encourage employees’ productivity and decrease turnover.

According to Mullins [2], there is a significant relationship between organizational climate and commitment of employees as well as perceived organizational performance. However, a healthy organizational climate does not guarantee an improved organizational performance, even along with organizational commitment, there are other variables contributing to improved performance [2].

### Organizational commitment

There have been various classifications of employee attitude and attachment towards their organizations such as loyalty, devotion and commitment in the extant literature. The concept of commitment was firstly introduced to literature in 1960 by Becker and it was explained as “one mechanism producing consistent human behavior” [13]. Later, during 1970s, a variety of studies were carried out on the concept of organizational commitment. One of the important studies of the literature was carried out in 1974 by Lyman Porter et al., which was concerned with organizational commitment and turnover intentions of the employees. Porter et al., in 1974, carried out the most commonly cited study of the related literature, which addressed the relationship between organizational commitment, job satisfaction and turnover intentions among a sample of psychiatric technicians. This study preserves its significance as it involves the first organizational commitment questionnaire in the literature [14]. Later in 1983, Morrow advocated that personal values, career, job (characteristics) and union play an important role in defining the commitment of workers [15]. Continuously, in 1985, Reichers introduced the idea that organizational commitment can be explained as “a collection of multiple commitments to various groups that comprise the organization” [16]. Later in 1991, Meyer and Allen adopted a different point of view and conceptualized the three component model of organizational commitment [17]. Allen and Meyer [17] explained the three components of organizational commitment as “Affective” (AC), “Continuance” (CC) and “Normative” (NC) commitment. In their conceptualizations, employees with higher levels of affective commitment remain in their organizations because they “want to”, those with strong continuance commitment levels stay in their organizations because they “need to” and those associated with normative commitment remain because they feel they “ought to” do so. According to the definition of Meyer and Allen, concept of organizational commitment is “a psychological state that characterizes the employee’s relationship with the organization and has implications for the decision to continue or discontinue membership in the organization” [17].

In the recent review of literature for antecedents and consequences of organizational commitment, the most encountered antecedents are found to be personal characteristics, organizational structure, tenure, rewards, training, and work values whilst the consequences are mainly increased employee performance, motivation and lower turnover intentions [18, 19]. The antecedent of organizational commitment in health sector is job motivation. Intrinsic motivation is found to lead to affective and normative commitment and extrinsic to normative commitment [20]. In addition, demographic characteristics like the marital status of workers and level of affective commitment and normative commitment are found to be positive and thus, it is found that there is a statistically significant difference in normative commitment levels in regard to the educational status of the respondents [21].

### Perceived organizational performance

In today’s competitive business environment, it is important for companies to perform better than the rival firms in any industry. The main task of an organization’s structure should serve an environment, which encourages people to work hard, and can coordinate their efforts to ensure higher levels of organizational performance [22]. Better performance depends on the overall performance of the organizations that is directly linked with human resources, in other words, employees. Although technology is important as it has a great impact on organizational performance in a number of ways, people are the necessary human resources whose knowledge and performance are important for advancing the purpose, mission, and strategies of an organization [23]. In short, organizational performance can be defined as the collective performance of individual employees whereas individual employee performance is defined as “an evaluation of the results of a person’s behavior: determining how well or poorly a person has accomplished a task given” and it is found that motivation, personality and ability are the important factors affecting employees’ performance [22]. Additionally, stress levels in an organization are found to be positively affecting organizational performance on the appropriate levels but when stress starts to increase, it leads to a decrease in individual and organizational performance [24].

Mostly, organizational performance is measured by evaluating the numerical data, which include the objective and timely information about how well the organization is doing. However, performance measurement is not always necessarily based on objective data. For example, customer satisfaction is measured according to individual stories and managers decide on how well an organization is doing according to those collected subjective data [25]. Perceived organizational performance varies according to whose viewpoint is taken within the organization such as customers or stockholders. The time period observed and criteria used also affect the perceived performance [26]. The concept of perceived organizational performance specifically refers to the subjective measurement of employee perceptions regarding an organization’s overall performance when compared to the rivals in the same sector and may be closely related to strategy and reward systems that directly affect the attitudes of employees within an organization [27]. In this sense, human resources practices in an organization surely contribute to overall organizational performance and efficiency since organizational performance can be defined as the individual perception of organizational efficiency by employees [28]. Continuously, in order to enhance organizational performance it is important for management to understand and find different sources of leadership that will lead to improved organizational performance [11]. Teamwork is also found to be one of the antecedents for organizational performance [23].

As a result of the literature review, the study has adopted four hypotheses to be tested:

#### H_1_:

There is a positive relationship between organizational climate and organizational commitment.

#### H_2_:

There is a positive relationship between organizational climate and perceived organizational performance.

#### H_3_:

Organizational climate is statistically significant in predicting organizational commitment.

#### H_4_:

Organizational climate is statistically significant in predicting perceived organizational performance.

## Methods

### Measurement

Data was collected with the help of self-administered surveys introduced to health care workers currently employed in four of the five largest public hospitals of North Cyprus. The collected data was evaluated and hypothesized model was tested with the help of Statistical Package for Social Sciences (SPSS) version 22. In order to test the hypothesized model of the study, correlation analysis and simple linear regression analyses were utilized.

The study aimed at testing the proposed model by considering the organizational climate as the only independent variable while organizational commitment and perceived organizational performance were considered as the dependent variables.

The data was collected with the help of self-administered questionnaires and evaluated by using correlations and simple linear regression in order to reveal the nature and power of the hypothesized relationship between variables. Before utilizing the recently mentioned tests, factor analysis and, consequently, reliability analysis were carried out for each part of the instrument in order to test the validity and reliability of the instrument for the current research setting.

The questionnaires involved 5-point Likert scale measures and were adopted from the studies mentioned below:

- Organizational Climate Scale (CLIOR) [29]
- Organizational Commitment Questionnaire [30]
- Perceived Organizational Performance [31]

Organizational Climate Scale (CLIOR) was adopted from the study of Peña-Suárez et al. [29]. The short version of the questionnaire was adopted for the present study, which included 15 items aimed to measure the organizational climate. The first 15 questions of the survey consisted of questions regarding organizational climate. However, 1 item was removed after the pilot study, which caused a problem with the reliability of the instrument (Cronbach’s Alpha for 15 items: 0.567 and Cronbach’s Alpha when one item was deleted: 0.884). Therefore, 14 items of the CLIOR Scale were included into the main study. Questions from Meyer and Allen’s [17] Organizational Commitment Questionnaire (OCQ) were adopted in order to measure the level of organizational commitment of the health care employees. There were 17 items aimed at measuring the organizational commitment score of the respondents. The overall organizational commitment score was taken as the average score of all three dimensions as suggested by Meyer and Allen [30]. On the other hand, questions from Delaney and Huselid’s [31] study regarding the perceived organizational performance were used to measure the overall score of perceived organizational performance.

All three measurement instruments were originally produced in English language so, for the current research, the items were translated into Turkish by using back- translation method in order to get the best response from the participants.

### Sampling

Currently, there are five large public hospitals in North Cyprus. Current study utilized the information, which was gathered from four large hospitals, and one hospital was not taken into the sample of the study. It was excluded from the scope of the study because of the communication issues and unwillingness of the staff in that public hospital. The universe of the study consisted of 975 health care employees who are currently employed full time in those four hospitals.

Simple random sampling method was used in order to select the participants for the study. When calculating the minimum sample for respondents, which was required to represent the universe, 90% confidence level and 5 confidence interval was considered for total population of *N* = 975 healthcare workers in four public hospitals. When the sample for the study was calculated, the required sample size for the current study was found to be 212 respondents.

Approximately 1000 questionnaires were distributed by hand, and around 220 were collected back. However, some of them were found to be inadequately answered, so they were left out of consideration. Finally, 213 returned questionnaires were used and the response rate was 21.8%.

The reliability of the measurement instruments was studied before moving on to the actual analysis of hypothesis testing. The minimum level of Cronbach’s Alpha was above 0.7 for all instruments.

## Results

### Descriptive findings

The majority of the study sample was female respondents (74.6%) and were between 31 and 40 years of age (40.6%). Due to the fact that it was easier to reach nurses in the hospital setting, the majority of the sample was found to be nurses (48.4%). Percentage of nurses was followed by a considerable amount of other health care employees like pharmacists, dieticians, radiology technicians, medical secretaries, physiotherapists, chemists, laboratory assistants and biologists (38%). Again, due to another important fact that it was very difficult to reach doctors in the hospital setting, the minority of the respondents was doctors (12.7%).

Lastly, when the frequency about tenure is reviewed, it is possible to conclude that there is an almost equal distribution of the tenure between 1 to 10 years of work. The majority mentioned that they have been working in the same institution for more than 10 years (31.5%). Remaining population stated that they have been in the same organization for 5 to 10 years (25.4%) and 1 to 5 years (21.1%). Only a minority of the population mentioned that they have been in the same organization for only less than 1 year (3.3%). Interestingly, 40 of the respondents (18.8%) rejected to state their tenure (Table 1).

**Table 1:** Demographic data of respondents (*N* = 213)

Variable	Frequency	Percentage (%)
Gender		
Female	159	74,6
Male	52	24,4
Missing	2	,9
Age		
Between 20 and 30	62	29,1
Between 31 and 40	86	40,4
Between 41 and 50	56	26,3
More than 50	7	3,3
Missing	2	,9
Profession		
Doctor / Practitioner	27	12,7
Nurse	103	48,4
Other	81	38,0
Missing	2	,9
Tenure		
Less than one year	7	3,3
1–5 Years	45	21,1
5–10 Years	54	25,4
More than 10 Years	67	31,5
Missing	40	18,8

### Correlation analysis

Subsequently, correlations were checked in order to confirm the hypothesized relationship between independent variables and dependent variables. When correlations are reviewed, it is possible to conclude that organizational climate is positively correlated with both organizational commitment and perceived organizational performance (Table 2).

**Table 2:** Intercorrelations among control variables, organizational climate, organizational commitment and perceived organizational performance

Variable	1	2	3	4	5	6	7	8	9
1. Organizational Climate	1000								
2. Organizational Commitment	0,452^a^	1000							
3. Perceived Organizational Performance	0,671^a^	0,436^a^	1000						
4. Gender	0,003	0,050	0,077	1000					
5. Age	0,053	0,174^b^	− 0,048	0,083	1000				
6. Marital Status	−0,041	− 0,020	− 0,022	0,113	− 0,094	1000			
7. Duty	−0,027	0,186^b^	− 0,097	−0,132	0,017	0,005	1000		
8. Salary	−0,163^b^	− 0,022	−0,205^a^	0,065	0,438^a^	− 0,014	−0,201^b^	1000	
9. Tenure	0,018	0,213^a^	− 0,082	−0,026	0,579^a^	− 0,149	0,218^a^	0,458^a^	1000

^a^Correlation is significant at the 0.01 level (2-tailed)

^b^Correlation is significant at the 0.05 level (2-tailed)

According to correlation coefficients of organizational climate and organizational commitment, these two concepts have a positive relationship, which is found to have a moderate strength level of 0.452 [32]. Therefore, it is possible to accept that there is a positive relationship between organizational climate and organizational commitment and thus, this finding supports Hypothesis 1.

When correlation coefficient of organizational climate and perceived organizational performance is reviewed, the relationship is found to be positive with a significantly high strength level of 0.671. Therefore, it is possible to accept that there is a positive relationship between organizational climate and perceived organizational performance, and Hypothesis 2 is supported with this finding.

As an overall result, it is concluded that organizational climate has a positive linear relationship with organizational commitment and perceived organizational performance on moderate to high level of strength in terms of correlation coefficients. After concluding that the relationship between variables is a linear relationship, a test of normality was also run in order to see the normal distribution before the simple linear regression. As a result, both organizational commitment and perceived organizational performance values were found to have normal distribution (sig. 0.200 > 0.05).

Additionally, control variables were included in the correlations matrix in order to comprehend the relationships between demographic variables and organizational climate, organizational commitment and perceived organizational performance.

According to the results from correlation analysis, organizational commitment and age have a weak, positive correlation at 0,05 significance level (0,174). This can be concluded as older employees tend to have higher levels of organizational commitment. Tenure and organizational commitment have a weak and positive relationship (0,213) meaning that as employees remain in the same organization for a longer period of time, their organizational commitment tends to increase. There is a positive but weak correlation between organizational commitment and duty (0,186), which can be interpreted as employees working in other positions (as pharmacists, pharmacist helpers, dieticians, radiology technicians etc.) besides doctors, nurses and managers, tend to have higher levels of organizational commitment.

Continuously, organizational climate was found to have a negative and weak correlation with salary (− 0,163). This result can represent that employees tend to observe the climate in their organizations less when they receive higher salaries.

Lastly, perceived organizational performance was also found to have a negative and weak correlation with salary (− 0,205). This result can be interpreted as employees tend to be concerned less about organizational performance when they receive higher salaries.

### Regression analysis

Due to the fact that the study adopted only one independent variable and aimed to find out the direct influence of organizational climate on the dependent variables without a confounding effect or variable, simple linear regression analysis was carried out. Initial regression analysis was conduced to test the relationship between organizational climate and organizational commitment in the first place.

Organizational climate was found to be statistically significant in predicting organizational commitment (0.000 < 0.05) so, Hypothesis 3 was supported (Table 3).

**Table 3:** Simple linear regression organizational climate and organizational commitment

	B	Std. Error	Beta	t	Sig.
Constant	1.695	.214		7.907	.000
Organizational Climate	.410	.062	.452	6.589	.000

Dependent Variable: Organizational Commitment R^2^: 0.204 Sig: < 0.05

Furthermore, as a result of the regression analysis, it is possible to predict the organizational commitment by organizational climate by the equation as follows:

$$ \mathrm{Organizational}\ \mathrm{commitment}=1.695+0.410\ \left(\mathrm{Organizational}\ \mathrm{climate}\right) $$

The predictive power of regression equation was found to be 0.204 meaning that 20.4% of organizational commitment’s variance can be predicted by organizational climate.

Continuously, second linear regression analysis was conducted in order to evaluate the relationship between organizational climate and perceived organizational performance. Organizational climate was found to be statistically significant in predicting perceived organizational performance (0.000 < 0.05) and Hypothesis 4 was supported (Table 4).

**Table 4:** Simple Linear Regression Organizational Climate and Perceived Organizational Performance

	B	Std. Error	Beta	t	Sig.
Constant	.471	.223		2.112	.036
Organizational Climate	.769	.065	.671	11.805	.000

Dependent Variable: Perceived Organizational Performance R^2^: 0.450 Sig: < 0.05

As a result of the regression analysis, it is possible to predict the perceived organizational performance by organizational climate by the equation as follows:

$$ \mathrm{Perceived}\ \mathrm{organizational}\ \mathrm{performance}=0.471+0.769\ \left(\mathrm{Organizational}\ \mathrm{climate}\right) $$

The prediction power of regression equation was found to be 0.450 meaning that 45% of perceived organizational performance’s variance can be predicted by organizational climate.

**Fig. 1:**
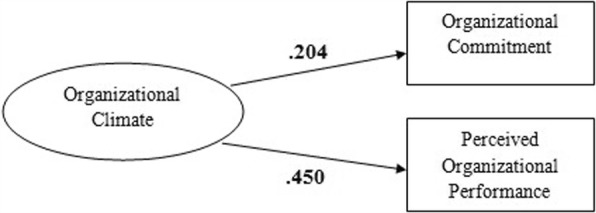
Tested model of the study

As a result of the regression analysis, it is possible to adjust the proposed model as follows: (Fig. 1).

The findings from the regression concluded that there is a strong influence of organizational climate on perceived organizational performance and a moderate influence on organizational commitment. By using the equations which were constructed by results of regression analysis, it is possible to predict how much influence does organizational climate have on the dependent variables, organizational commitment and perceived organizational performance, which is present as 20.4% and 45% respectively.

## Discussion

The results of the study provided critical information regarding the influence of organizational climate on organizational commitment and perceived organizational performance in four largest public hospitals included in the study. Results from the regression analysis suggested that organizational climate has an impact on predicting organizational commitment and perceived organizational performance of the employees in public hospitals of North Cyprus. Organizational climate is found to be statistically significant in determining the organizational commitment of the employees. Continuously, there was a positive and linear relationship between these two variables. This can be interpreted as if the organizational climate scores of the employees are high, organizational commitment scores of the employees are high at the same time. In other words, if the employees in public hospitals of North Cyprus perceive the organizational climate in a positive way, they will have higher levels of organizational commitment. However, the predictive power of organizational climate on organizational commitment is not significantly high by 20%, which means that the organizational climate is only effective in predicting organizational commitment by 20%.

On the other hand, the prediction power of the organizational climate on perceived organizational performance is found to be significantly high by - 45%, which means that if employees perceive a positive organizational climate in their organizations, they tend to perceive the organizational performance higher in comparison to competitors.

Consequently, in order to reach positive levels of organizational climate in hospital settings, it is important for decision makers to understand and improve the components of organizational climate.

## Conclusion

Firstly, it is important to create an environment of trust involving good relationships with supervisors to contribute to the organizational commitment of the employees and improve their perception of organizational performance. Employees should be openly communicating with their supervisors and receiving feedback and support when needed. In addition, adopting work teams can improve the work environment by constituting a warm and cooperating atmosphere while reducing the conflict. Continuously, autonomy is another important aspect where employees take full responsibility of their jobs and can take initiatives. At this point, practicing in participative management will be useful for the decision makers so that they share the authority with the employees allowing them to take more responsibilities, which will contribute, to their commitment and perceptions.

Secondly, the perception of employees on equality and fairness in distribution of rewards is also crucial. Managers should offer rewards to their employees and at the same time watch over the distribution of rewards to create a positive climate because a perception of unfairness can cause a reverse impact.

Thirdly, organizational structure, including regulations, standards and organization of work, is also another component of organizational climate, which has an impact on organizational commitment and perceived organizational performance. It is not only important for the patients but also for the physical conditions of employees. In order to create a positive organizational climate, decision makers can transform the current mechanistic structure of the public hospitals into more of an organic structure, which includes flexibility and decentralization.

Lastly, organic structures can also contribute to openness to innovation and reduce the resistance to change. Considering that organic structures are sensitive to the external environment, they are always open to change and encourage employees to be creative and innovative. So then, organizational climate can be perceived more positively and organizational commitment and perceived organizational performance will be affected positively.

### Limitations

Although the research has successfully reached its aim of analyzing the influence of organizational climate on commitment and perceived performance, there were some limitations, which cannot be disregarded.

Current study tried to reach a sample size, which would produce more reliable results that can be generalized to the universe of the study. Thus, as a result of the communication problems and unwillingness of the staff in one hospital, that hospital had to be taken out of the sample, which is the most important limitation of the study.

In addition, the model of the study did not concentrate on the relationship between organizational commitment and perceived organizational performance.

Continuously, there was no analysis regarding a confounding variable. Therefore, a mediating or moderating relationship was not sought for organizational commitment in respect to the influence of organizational climate on perceived organizational performance.

## Abbreviations

CLIOR: Organizational Climate Scale
OCQ: Organizational Commitment Questionnaire
SPSS: Statistical Package for Social Sciences
